# Heliconiini butterflies can learn time-dependent reward associations

**DOI:** 10.1098/rsbl.2020.0424

**Published:** 2020-09-23

**Authors:** M. Wyatt Toure, Fletcher J. Young, W. Owen McMillan, Stephen H. Montgomery

**Affiliations:** 1 Department of Biology, McGill University, 1205 Docteur Penfield, Montreal, Canada H3A 1B1; 2 Smithsonian Tropical Research Institute, Gamboa, Panama; 3 Department of Zoology, University of Cambridge, Downing Street, Cambridge, CB2 3EJ, UK; 4 School of Biological Science, University of Bristol, 24 Tyndall Avenue, Bristol, BS8 1TQ, UK

**Keywords:** contextual learning, circadian memory, cognitive ecology, Lepidoptera

## Abstract

For many pollinators, flowers provide predictable temporal schedules of resource availability, meaning an ability to learn time-dependent information could be widely beneficial. However, this ability has only been demonstrated in a handful of species. Observations of *Heliconius* butterflies suggest that they may have an ability to form time-dependent foraging preferences. *Heliconius* are unique among butterflies in actively collecting pollen, a dietary behaviour linked to spatio-temporally faithful ‘trap-line' foraging. Time dependency of foraging preferences is hypothesized to allow *Heliconius* to exploit temporal predictability in alternative pollen resources. Here, we provide the first experimental evidence in support of this hypothesis, demonstrating that *Heliconius hecale* can learn opposing colour preferences in two time periods. This shift in preference is robust to the order of presentation, suggesting that preference is tied to the time of day and not due to ordinal or interval learning. However, this ability is not limited to *Heliconius*, as previously hypothesized, but also present in a related genus of non-pollen feeding butterflies. This demonstrates time learning likely pre-dates the origin of pollen feeding and may be prevalent across butterflies with less specialized foraging behaviours.

## 1. Introduction

The foraging ecology of a species shapes which environmental cues reliably predict resource availability, and this can subsequently influence what association foragers can easily make [1]. For example, wild *Drosophila* forage on rotting fruits [2] and therefore readily learn associations with odour cues, but in artificial environmental contexts where colour cues are reliable and odours are not, *Drosophila* evolve strong visual learning propensities [3].

For many pollinators, foraging for flowers occurs in the environmental context of temporal variation in resource profitability. Flowers tend to vary predictably in the temporal availability of pollen and nectar [4]. Consequently, some specialist nectarivores use time as a contextual cue to modulate their foraging strategy [5]. For example, honeybees can consistently change their preference towards particular visual cues throughout the day [6,7], and some nectarivorous ants remember the time of day at which resources are most profitable [8,9]. However, the ability to learn time-dependent associations has only been demonstrated in a handful of insects, including fruit flies, bees and ants [7,10–15], and there is evidence that this ability can vary across species from the same family [16,17]. Hence, the prevalence of this ability, and the foraging traits that may account for its variability, are unclear.

Butterflies of the genus *Heliconius* actively collect and feed on pollen grains, a foraging behaviour unique among the 17 000+ described species of butterfly [18–20]. *Heliconius* collect pollen from a restricted range of plants that occur in low densities, but vary predictably in their timing of pollen release and nectar production [18,21,22]. Pollen feeding is associated with a suite of derived foraging adaptations not seen in other tropical butterflies, including fidelity to a local home-range [21,23], and spatio-temporally faithful ‘trap-lining' behaviour, whereby individual butterflies consistently visit particular flowers at specific times of day [21]. Although data are limited, these behaviours are thought to be absent in non-pollen feeding Heliconiini [24].

Given their derived foraging behaviour, it has been hypothesized that *Heliconius* have evolved specific cognitive traits that support trap-lining behaviour, including the ability to use the time of day as a contextual cue [21,25]. However, to our knowledge, time-dependent associative learning has not been reported in any Lepidoptera. Additionally, whether this ability would be seen in non-pollen feeding species without foraging specializations such as trap-lining is not clear. In this study, we provide the first evidence that *Heliconius* butterflies can form time-dependent preferences for distinctly coloured flowers. However, we also find evidence that *Dryas iulia,* which does not pollen feed, can learn temporal information.

## 2. Materials and methods

Animal husbandry is described in the electronic supplementary material. We tested two *Heliconius* species (*H. hecale* and a smaller sample of *H. melpomene*) and one non-*Heliconius* species, *D. iulia*. Butterflies were trained on yellow or purple artificial feeders that contained either a 10% sugar solution with 2.5 CCF per 50 ml of critical care formula (surrogate for pollen; rewarded feeder), or a saturated quinine water solution (punished feeder). Purple and yellow were chosen as these colours are equally preferred by the species we tested (electronic supplementary material). Twelve feeders of each colour were placed on a grid of 24, with randomized positions (electronic supplementary material, figure S1). Butterflies were trained and tested in groups of 8–12 individuals and presented with feeders for 2 h in the morning (AM) (08.00–10.00) and 2 h in the afternoon (PM) (15.00–17.00).

### (a) Experimental procedure

The experiment had four phases. (i) During pre-training, butterflies were fed on white feeders, in the AM and PM, for 2 days. (ii) The naive shift in colour preference based on time of day was recorded prior to training, using clean, empty feeders. Due to low feeding rates in the PM session, we split the initial preference test across 2 days. AM colour preference was recorded on day 1, and butterflies were food deprived in the PM. PM colour preference was tested on day 2, after food deprivation in the AM. (iii) The training reward structure was split such that the yellow feeders were rewarded in AM and purple feeders rewarded in PM, or vice versa. This training phase lasted for 10 days. (iv) During the post-training preference tests butterflies were presented with clean, empty feeders for 1 h in the AM, followed by the reinforced AM feeders for 1 h, and then clean, empty feeders for an hour in the PM. To determine whether butterflies were learning the order in which they encountered the reward, rather than the time of day, half of the *H. hecale* had the order of their AM and PM trials reversed (see electronic supplementary material). During trials, feeders were filmed with a GoPro HERO-5 camera (electronic supplementary material, figure S1). Using this footage, we scored the number of feeding attempts made by each individual.

### (b) Training criterion

For an observed behaviour to be a consequence of learning, an animal must experience the reward contingency scheme [26]. Some individuals (*n* = 18) either did not attempt to feed on both feeders in AM or, more commonly, PM during training, or did not make any foraging attempts during a final test session and were removed from further analyses. Following previous learning studies [27–29], we also established a training criterion. As we are interested in whether time-dependent memories are formed and can therefore guide behaviour in the absence of the reward, we identified individuals that correctly adjust their behaviour in AM and PM sessions during training with reinforced feeders. We then asked whether these individuals demonstrate evidence of learning by behaving in the same way when presented with unreinforced feeders in the post-training preference test. Our training criteria was that the majority (greater than 50%) of feeding choices made by an individual in the final two training days were correct in both AM and PM.

### (c) Statistical analysis

Data were analysed using generalized linear mixed models (GLMMs) in R [30]. We asked whether the time of day influenced: (i) shifts in proportional preference for the colour rewarding in the morning when naive, using a binomial GLMM with response variable ‘morning reward colour choices/afternoon reward colour choices' and fixed factor ‘time of day' (morning or afternoon); (ii) shifts in proportional colour preference when trained, using the same specifications but with the additional fixed factor ‘presentation order' (standard or reversed). Identity was included as a random effect. The electronic supplementary material contains details of the full dataset and assumption checking.

## 3. Results

### (a) *Heliconius* can learn time-dependent associations

Across the *H. hecale* that experienced the full training set, there was no significant effect of the time of day on naive colour preferences (*z* = 0.90, *n* = 30, *p* = 0.36), and no overall effect of time of day on trained colour preference (*z* = −1.846, *n* = 30, *p* = 0.06). There was considerable variation in behaviour during training, and only a subset of individuals (*n* = 16) passed the training criterion (electronic supplementary material, figure S2). Prior to training, both butterflies that met the training criterion, and those that did not, showed no significant shift in colour preference throughout the day (*z* = 0.33, *n* = 16, *p* = 0.73, and *z* = 1.15, *n* = 14, *p* = 0.24, respectively).

**Figure 1.**
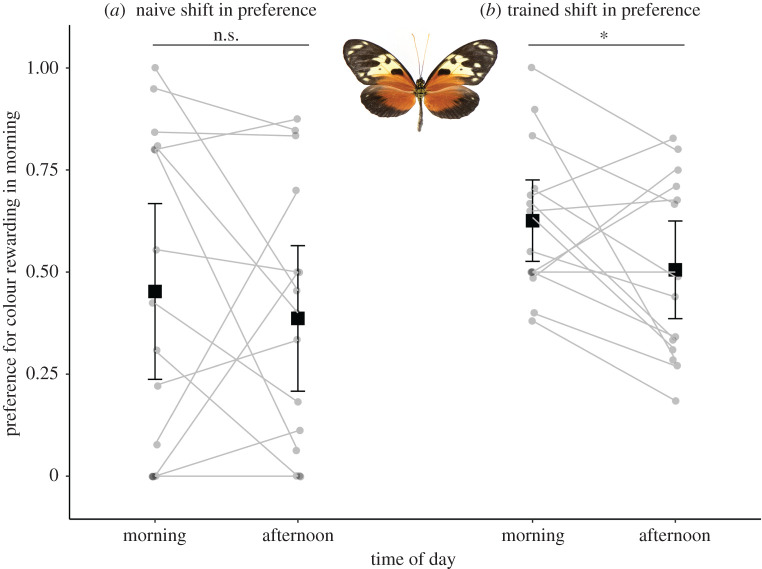
Data from colour preference trials of *H. hecale* meeting the training criterion. (*a*) Naive preferences in the morning and afternoon. (*b*) Preferences of butterflies from (*a*) post-training. Grey lines connect individuals. Data are means ± 95% CI. *p < 0.05.

However, after training, individuals that passed the training criteria learned to shift colour preference between the AM and PM (*z* = −2.24, *n* = 16, *p* = 0.02, figure 1*b*). On average, the preference for AM rewarded colour decreased by 11% from AM to PM. The presentation order of the post-training preference test (AM first versus PM first) had no effect (*z* = 0.36, *p* = 0.71, *n* = 16). Among individuals that did not meet the training criterion there was no shift in colour preference throughout the day after training (*z* = 1.05, *n* = 14, *p* = 0.29). Adding a smaller sample of *H. melpomene* (*n* = 6) supported and strengthened these results. We found no evidence to suggest social interactions influenced our results (see electronic supplementary material).

### (b) Evidence time learning is common across Heliconiini

**Figure 2.**
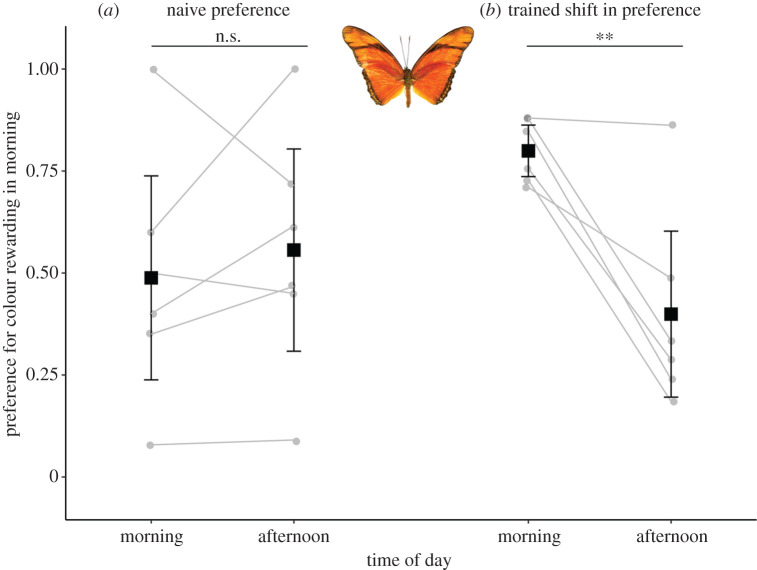
Data from colour preference trials of *D. iulia* meeting the training criterion. (*a*) Naive preferences in the morning and afternoon. (*b*) Preferences of butterflies from (*a*) post-training. Grey lines connect individuals. Data are means ± 95% CI. **p < 0.01.

In a secondary experiment using *Dryas iulia*, a closely related genus within Heliconiini that does not pollen feed, 12 individuals experienced the full training set, with no effect of time of day on trained colour preference (*z* = 0.01, *n* = 12, *p* = 0.99). However, consistent with data from *H. hecale*, variation in the behaviour during training resulted in only a subset of individuals (*n* = 6) passing the training criterion. Among these individuals, there was no significant effect of time of day on naive preference (*z* = 1.67, *n =* 6, *p* = 0.09), but post-training there was a significant effect of time on colour preference, with a preference for the AM rewarded colour decreasing on average by 40% from AM to PM (figure 2*b*, *z* = −9.334, *n* = 6, *p* < 0.001). Individuals that did not reach the training criterion did not shift colour preference between AM and PM before training (*z* = 0.437, *n* = 6, *p* = 0.66) and show a significant shift in the incorrect direction post-training (*z* = 7.354, *n* = 6, *p* < 0.001).

## 4. Discussion

We demonstrate that *Heliconius* butterflies can use time as a context for making foraging decisions. The observed shift in preference is similar in magnitude to observed temporal variation in floral visits by wild *Heliconius* [22]*.* Time-dependent learning can occur via an ordinal, interval or circadian timing mechanism [31]. Given presentation order has no effect on our results, we find no support for ordinal or interval learning as an explanation. While suggestive of circadian memory, our data do not confirm an endogenous mechanism, as it is possible butterflies are responding to external cues that covary with the time of day (e.g. light-levels, sun position). To our knowledge, these results provide the first experimental evidence of time-dependent learning in Lepidoptera.

Effectively obtaining pollen is important for fitness in *Heliconius* as it provides a reliable source of protein, leading to a pronounced delay of reproductive senescence and increased lifespan compared to other butterflies [32]. *Heliconius* have been observed foraging early in the morning to actively defend flowers against other butterflies and timing floral visits to periods of maximal pollen and nectar reward [18,22], suggesting selection may have favoured cognitive mechanisms that increase foraging efficiency. On this basis, it has been suggested that *Heliconius* may have acquired the ability to use time as a foraging cue in the context of pollen feeding [21]. Our experiments confirm that *Heliconius* can use time as a foraging cue but also show that a close, non-pollen feeding relative, *Dryas iulia*, has the same capacity. Although our sample size for *Dryas* is smaller than for *Heliconius*, the proportion of individuals passing the training criterion and the pattern of results are highly consistent with *Heliconius*.

It is notable that some individuals do not pass the training criteria or learn the association. While this is consistent with other butterfly learning experiments [33], it would be interesting to investigate whether variation in body condition upon emergence or alternative foraging tactics anecdotally observed in wild *Heliconius*, such as stationary feeding from restricted plant clusters versus trap-line foraging from broad arrays of diverse plants [18] contribute to the variability observed in passing the training criterion. Regardless, our data indicate both *Dryas* and *Heliconius* can learn time-dependent associations. This strongly suggests time learning pre-dates the origin of pollen feeding in *Heliconius,* and most likely did not evolve in response to selection for trap-line foraging. While data on the foraging behaviour of *Dryas* in the wild are limited, they have no known foraging specializations beyond those seen in other butterflies [34]. The ability to use time as a contextual foraging cue may therefore be widespread across nectarivorous butterflies.

Time learning is also notably prevalent among social Hymenoptera, where allocentric foraging provides an ecological context for using time cues in the context of a consistent foraging landscape [35,36]. *Heliconius* have converged on several foraging behaviours observed in these species and also share dramatically expanded mushroom bodies, a region of the insect brain responsible for learning and memory [37]. The ecological challenges associated with learning foraging sites likely exert selective pressures favouring neuroanatomical elaboration that supports specialized cognitive abilities, like time learning [5,38–41]. However, our data on *Dryas* suggest that elaborated mushroom bodies are not necessary for time learning. This is further supported by the fact that *Drosophila*, which have substantially smaller mushroom bodies, can also learn time-dependent olfactory associations [10]. Integrating time and place memories may be more complex than forming these associations in isolation, as hypothesized in hummingbirds [38,42]. However, time learning is likely to be an important precursor for temporally and spatially faithful foraging. Hence, the pre-existence of this trait may have helped facilitate the evolution of trap-lining, and the transition to pollen feeding in *Heliconius*. Overall, our results support the importance of temporal predictability in resources, rather than pollen feeding or allocentric foraging *per se*, in promoting ability for time learning.

## Supplementary Material

Additional results for Heliconiini butterflies learn time-dependent reward associations

## Data accessibility

Data and R scripts are available from the Dryad Digital Repository: https://doi.org/10.5061/dryad.c59zw3r4m [42].
